# The complete chloroplast genome of Korean cultivar ‘Harmony’ (*Prunus salicina* × *Prunus armeniaca*)

**DOI:** 10.1080/23802359.2022.2132839

**Published:** 2022-10-20

**Authors:** Se Hee Kim, Jung-hyun Kwon, Kang Hee Cho, Ji Hae Jun, Il Sheob Shin

**Affiliations:** a Fruit Research Division, National Institute of Horticultural and Herbal Science, Wanju, Republic of Korea; b Pear Research Institute, National Institute of Horticultural and Herbal Science, Naju, Republic of Korea

**Keywords:** Plumcot, Korean cultivar, Harmony, chloroplast genome

## Abstract

*Prunus* Linnaeus 1753 species include many economically important fruit crops, of which plumcot is generated by inter-species crossing between *Prunus salicina* Lindley 1830 × *Prunus armeniaca* Linnaeus 1753. In this study, the complete chloroplast genome of Korean plumcot cultivar ‘Harmony’ was characterized through the *de novo* assembly of Nanopore and Illumina sequencing data. The chloroplast genome is a circular molecule of 157,916 bp length with 36.75% GC content and has a total of 113 genes including 79 protein-coding genes, 30 transfer RNA genes, and four ribosomal RNA genes. Phylogenetic analysis with protein-coding sequences of chloroplast genome revealed that ‘Harmony’ showed closer to *P*. *salicina* than *P*. *armeniaca.*

‘Harmony’ is a plumcot originating from a cross between ‘Soldam’ plum (*Prunus salicina* Lindley 1830) and ‘Harcot’ apricot (*Prunus armeniaca* Linnaeus 1753) made in 1999 at the National Institute of Horticultural and Herbal Science (NIHHS) of the Rural Development Administration (RDA) in Korea. After further evaluation of its characteristics, interspecific hybrid was named as ‘Harmony’ in 2007 (Jun et al. 2011). ‘Harmony’ was cultivated in the experimental field of NIHHS (35°50′42ʺN, 127°8′51ʺE) and its voucher specimen (IT253867) was registered at National Agrobiodiversity Center of the RDA (http://genebank.rda.go.kr/eng/uat/uia/actionMain.do, Mun-sup Yoon, msyoon63@korea.kr). Chloroplast genomes are important sources for phylogenetic analyses, genetic diversity evaluation and plant molecular identification (Sun et al. 2020). Genomic DNA was extracted from leaves using Plant gDNA extraction kit (Biomedic, Korea). Nanopore sequencing library was constructed using the ONT 1D ligation sequencing kit (SQK-LSK110, Oxford, UK) and sequenced using GridION platform. Illumina paired-end (PE) library was constructed using TruSeq DNA PCR-Free kit (Illumina, San Diego, CA) and sequenced using the Illumina HiSeqX platform. Raw sequencing data of Nanopore 12.0 Gb and Illumina 8.4 Gb were generated and trimmed using Porechop ver. 0.2.3 (https://github.com/rrwick/Porechop) and quality_trim program in CLC Assembly Cell package ver. 4.2.1 (QIAGEN, Aarhus, Denmark), respectively. Trimmed Nanopore data were *de novo* assembled using NextDenovo ver. 2.3.1 (https://github.com/Nextomics/NextDenovo) and then chloroplast contigs were selected by similarity searches with reported *Prunus* chloroplast genomes (NC_047442.1 and MW406469.1). The selected contigs were polished using NextPolish ver. 1.3.1 (https://github.com/Nextomics/NextPolish) and then manually error-corrected by mapping trimmed Illumina data using clc_ref_assemble program in the CLC Assembly Cell package ver. 4.2.1. The contigs were merged and gap-filled to generate complete chloroplast genome. Sequence error in the genome was checked and corrected again by read mapping and manual curation. The complete chloroplast sequence was annotated using the GeSeq (Tillich et al. 2017) and Artemis (Carver et al. 2012) programs with reported chloroplast genomes in *Prunus* genus. In addition, the precise gene regions were determined by manual curation using BLAST searches.

Chloroplast genome of ‘Harmony’ is a circular molecule of 157,916 bp length with 36.75% GC content (GenBank accession number MZ647490). The genome consists of a pair of inverted repeats of 26,382 bp, a large single-copy of 86,123 bp and a small single-copy of 19,029 bp. A total of 113 genes were predicted in the genome including 79 protein coding genes, 30 transfer RNA genes, and four ribosomal RNA genes. The genome sequence showed 100% and 99.5% similarity with those of *P*. *salicina* (NC_047442, 157,916 bp, Xue et al. 2019) and *P*. *armeniaca* (KY101151, 157,951 bp), respectively.

Phylogenetic analysis of ‘Harmony’ with other *Prunus* species was performed using a maximum-likelihood method with conserved protein-coding sequences and revealed that ‘Harmony’ was phylogenetically closer to *P*. *salicina* than *P*. *armeniaca* (Figure 1). This indicated that the chloroplast of *P*. *salicina* was maternally inherited to ‘Harmony’, while nuclear genome sequences of ‘Harmony’ were from both parents (Supplementary Material 1). The complete chloroplast genome sequence data generated in this study will provide useful genetic information for understanding the phylogenetic relationships within the *Prunus* species at a molecular level.

**Figure 1.**
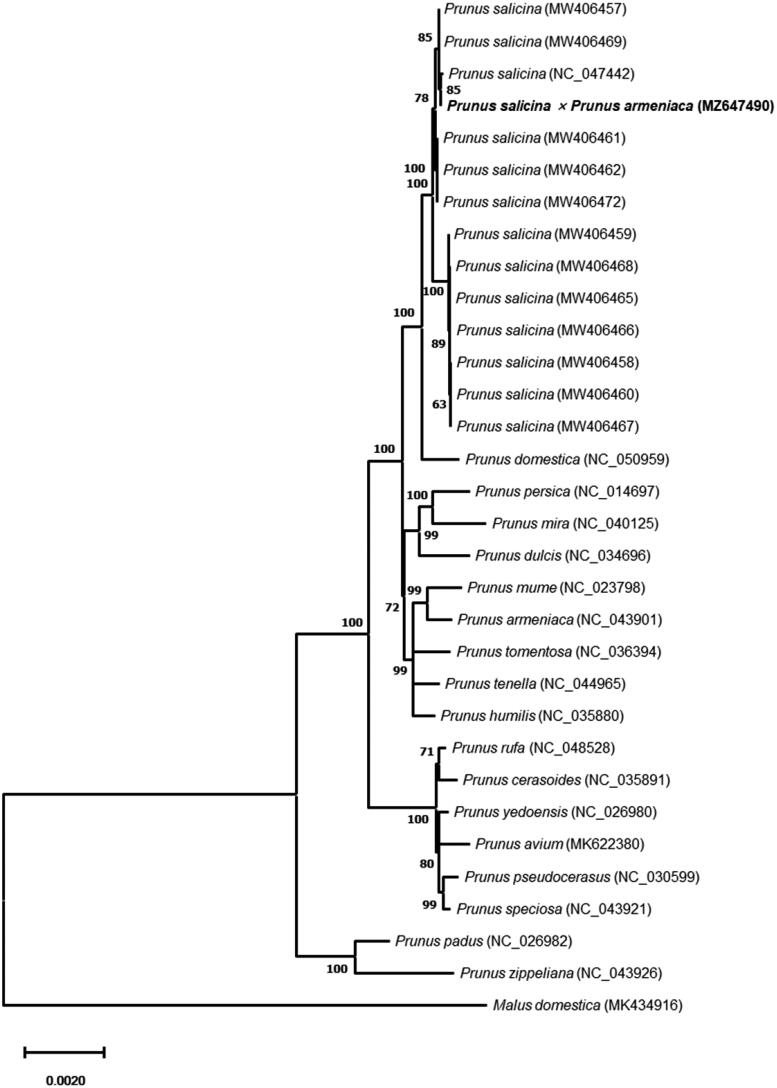
Maximum-likelihood phylogenetic tree of chloroplast genomes of Harmony and its related species. A total of 74 protein-coding sequences conserved in the chloroplast genomes of 32 species were multiple-aligned using MAFFT (http://mafft.cbrc.jp/alignment/server/index.html) and used to generate phylogenetic tree by MEGA 11 (Tamura et al. 2021). The bootstrap support values (>50%) from 1000 replicates are indicated on the nodes. GenBank accession nos. of chloroplast genome sequences used for this tree are indicated within parentheses.

## Supplementary Material

Supplemental Material

## Data Availability

The genome sequence data that support the findings of this study are openly available in GenBank of NCBI at https://www.ncbi.nlm.nih.gov under the accession no. MZ647490. The associated BioProject, Bio-Sample, and SRA numbers are PRJNA749137, SAMN20354921 and SRR15221231 (Illumina data), and SRR15221232 (Nanopore data), respectively.
