# Supplementary material for: The complete chloroplast genome of Korean cultivar ‘Harmony’ (Prunus salicina × Prunus armeniaca)
Source: Mitochondrial DNA B Resour. 2022 Oct 20;7(10):1820–2. doi: 10.1080/23802359.2022.2132839 (PMC9586692; doi:10.1080/23802359.2022.2132839)

**ONLINE SUPPLEMENTARY MATERIAL**

Supplementary Material 1. Mapping results of Illumina sequencing data to ITS1-5.8S-ITS2 sequences of *Prunus salicina* and *Prunus armeniaca.* (A) Comparison of ITS1-5.8S-ITS2 sequences of *P. salicina* (GenBank Accession no. AF185618.1) and *P. armeniaca* (GenBank Accession no. AF185620.1). Sequence alignment was performed and visualized using MultAlin tool (<http://multalin.toulouse.inra.fr/multalin/>) with default parameters. (B) Depth graph of mapped reads on *P. salicina* ITS1-5.8S-ITS2 sequences. (C) Depth graph of mapped reads on *P. armeniaca* ITS1-5.8S-ITS2 sequences. Illumina sequencing data of ‘Harmony’ were mapped on the ITS1-5.8S-ITS2 sequences and depth of mapped reads was calculated respectively using clc_ref_assemble and clc_mapping_info with default parameters in CLC Assembly Cell package ver. 4.2.1 (QIAGEN, Denmark). This result indicates that ITS1-5.8S-1TS2 sequences of both parents are present in ‘Harmony’, and thus supports that ‘Harmony’ used in this study is a hybrid of *P. salicina* × *P. armeniaca*.


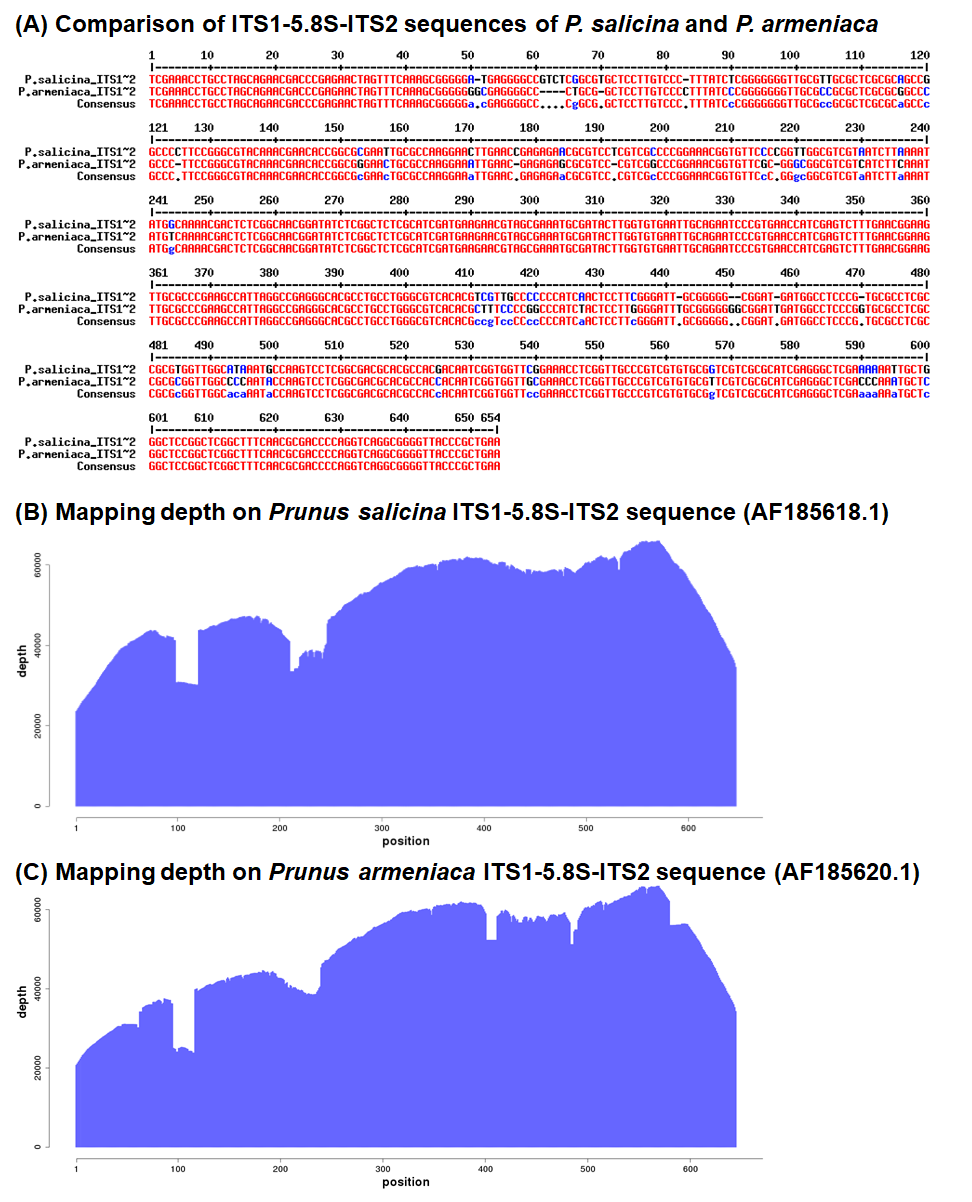

Supplement: Supplemental Material [file TMDN_A_2132839_SM7421.docx]
